# Targeted delivery of a vaccine protein to Langerhans cells in the human skin via the C‐type lectin receptor Langerin

**DOI:** 10.1002/eji.202149670

**Published:** 2022-01-09

**Authors:** Lydia Bellmann, Helen Strandt, Claudia Zelle‐Rieser, Daniela Ortner, Christoph H. Tripp, Sandra Schmid, Julia Rühl, Giuseppe Cappellano, Sandra Schaffenrath, Anastasia Prokopi, Sarah Spoeck, Athanasios Seretis, Barbara Del Frari, Stephan Sigl, Johanna Krapf, Christine Heufler, Tibor Keler, Christian Münz, Nikolaus Romani, Patrizia Stoitzner

**Affiliations:** ^1^ Department of Dermatology Venereology and Allergology Medical University of Innsbruck Innsbruck Austria; ^2^ Institute of Experimental Immunology University of Zürich Zürich Switzerland; ^3^ Department of Health Sciences Interdisciplinary Research Center of Autoimmune Diseases Center for Translational Research on Autoimmune and Allergic Disease‐CAAD Università del Piemonte Orientale Novara Italy; ^4^ Research Institute for Biomedical Aging Research University of Innsbruck Austria; ^5^ Department of Plastic Reconstructive and Aesthetic Surgery Medical University of Innsbruck Innsbruck Austria; ^6^ Celldex Therapeutics Hampton New Jersey USA

**Keywords:** immunotherapy, Langerhans cells, Langerin, skin dendritic cells, vaccination approaches

## Abstract

Human skin is a preferred vaccination site as it harbors multiple dendritic cell (DC) subsets, which display distinct C‐type lectin receptors (CLR) that recognize pathogens. Antigens can be delivered to CLR by antibodies or ligands to boost antigen‐specific immune responses. This concept has been established in mouse models but detailed insights into the functional consequences of antigen delivery to human skin DC in situ are sparse. In this study, we cloned and produced an anti‐human Langerin antibody conjugated to the EBV nuclear antigen 1 (EBNA1). We confirmed specific binding of anti‐Langerin‐EBNA1 to Langerhans cells (LC). This novel LC‐based vaccine was then compared to an existing anti‐DEC‐205‐EBNA1 fusion protein by loading LC in epidermal cell suspensions before coculturing them with autologous T cells. After restimulation with EBNA1‐peptides, we detected elevated levels of IFN‐γ‐ and TNF‐α‐positive CD4^+^ T cells with both vaccines. When we injected the fusion proteins intradermally into human skin explants, emigrated skin DC targeted via DEC‐205‐induced cytokine production by T cells, whereas the Langerin‐based vaccine failed to do so. In summary, we demonstrate that antibody‐targeting approaches via the skin are promising vaccination strategies, however, further optimizations of vaccines are required to induce potent immune responses.

## Graphical abstract

Antigen delivery to skin dendritic cells can be achieved by targeting either Langerin or DEC‐205 expressed on Langerhans cells and dermal dendritic cells. This study demonstrates that Langerin delivers proteins to Langerhans cells in vitro, however, DEC‐205 was more potent as vaccination approach when tested in a skin explant model.

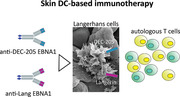

## Introduction

Dendritic cells (DC) fulfill a key role in immune surveillance by internalizing antigens and initiating antigen‐specific responses [1]. Whereas immune checkpoint inhibitors unleash pre‐existing T cells [2], DC with their unique capabilities can do both: boost pre‐existing immunity and induce de novo T cell responses, which makes them attractive targets for cancer immunotherapy [3, 4]. Their use in clinical trials also proved their safety with minimal side‐effects [5, 6, 7]. Obtaining large numbers of these cells is difficult, thus, methods were developed to target DC in vivo. Pioneering work from the group of Ralph Steinman performed in mice [8, 9] proved that antigen delivery to DC subsets in vivo by targeting antigens to surface receptors is a promising vaccination strategy. Particularly, various DC subsets can be selectively targeted with this method, as they express defined endocytic receptors [10]. Of them, the most exploited ones are C‐type lectin receptors (CLR), such as DEC‐205 (CD205), CLEC9A (CD370), and Langerin (CD207), resulting in antitumor/antiviral immunity in mouse models [11, 12, 13, 14]. To study translation into the human situation humanized mice were used, that is, CLEC9A [15], DEC‐205 [16], and DC‐SIGN [17]. In addition, antigen was successfully delivered to human DC by targeting DC‐SIGN [18], DEC‐205 [19, 20], and mannose receptor (MR) [21] in vitro and even in vivo into human subjects via DEC‐205 and MR resulting in antigen‐specific T‐cell responses [22, 23]. The first clinical trials of in vivo targeting with a cancer germ line/testis antigen coupled to an anti‐DEC‐205 antibody, CDX‐1401, induced some humoral and cellular immunity in patients with solid tumors with no signs of toxicity [5, 23]. These vaccines were given via intracutaneous injection, however, the functional capabilities of the addressed skin DC were not studied [5, 23].

The skin contains multiple DC subsets, such as dermal conventional DC1 (cDC1), cDC2, and Langerhans cells (LC), with different functional properties [24, 25]. These DC subsets express defined CLR [26] and can be targeted with specific antibodies or ligands [27, 28, 29]. For example, in a human skin explant model, the intradermal delivery of an anti‐DEC‐205 antibody resulted in binding according to CLR expression profiles, namely DEC‐205‐positive LC and dermal CD1a^+^ DC, whereas an anti‐Langerin antibody was found exclusively in migratory LC [27]. In this setting, T cell responses were not studied. However, to harness the potential of skin DC it is required to closely examine the T cell responses induced by in situ delivery of antibody‐antigen fusion proteins to human skin DC. This was the purpose of the study presented here. Herein, we demonstrate that an anti‐Langerin antibody can deliver protein antigen to LC and elicit T cell responses, however, the vaccine needs further optimization to achieve improved T cell immunity.

## Results

### Human antibodies are feasible for antigen targeting

In a clinical setting, targeting antibodies need to be of fully human origin [5, 23]. We, therefore, tested human anti‐human DEC‐205 and MR antibodies. Similar to the mouse targeting antibodies [27], intradermal injection of human anti‐human DEC‐205 and MR antibodies into healthy human skin explants showed that both were transported out of the skin tissue by migratory DC—an essential property for successful immunization. The defined subsets of skin DC were targeted differentially. In contrast to a control antibody of irrelevant specificity of the same isotype as anti‐DEC‐205, human anti‐human DEC‐205 antibody was bound and transported preferentially by CD1a^high^ LC and CD1a^+^ dermal cDC2. Expectedly, the CD14^+^ dermal macrophage‐like cells [30] were not targeted by anti‐DEC‐205 but rather by the antibody to MR, whereas LC were not targeted at all by anti‐MR antibody (Supporting information Figure S1).

These data recapitulate our preceding observations with mouse anti‐human DEC‐205 antibody [27]. Furthermore, they highlight the cellular events in the skin that explain the observed immunological responses in patients [5, 23] and they support the concept of antigen targeting. Hence, we conclude that human anti‐human targeting antibodies are suitable for clinical use.

### The cloned anti‐Langerin‐EBNA1 fusion protein binds to LC

**Figure 1:**
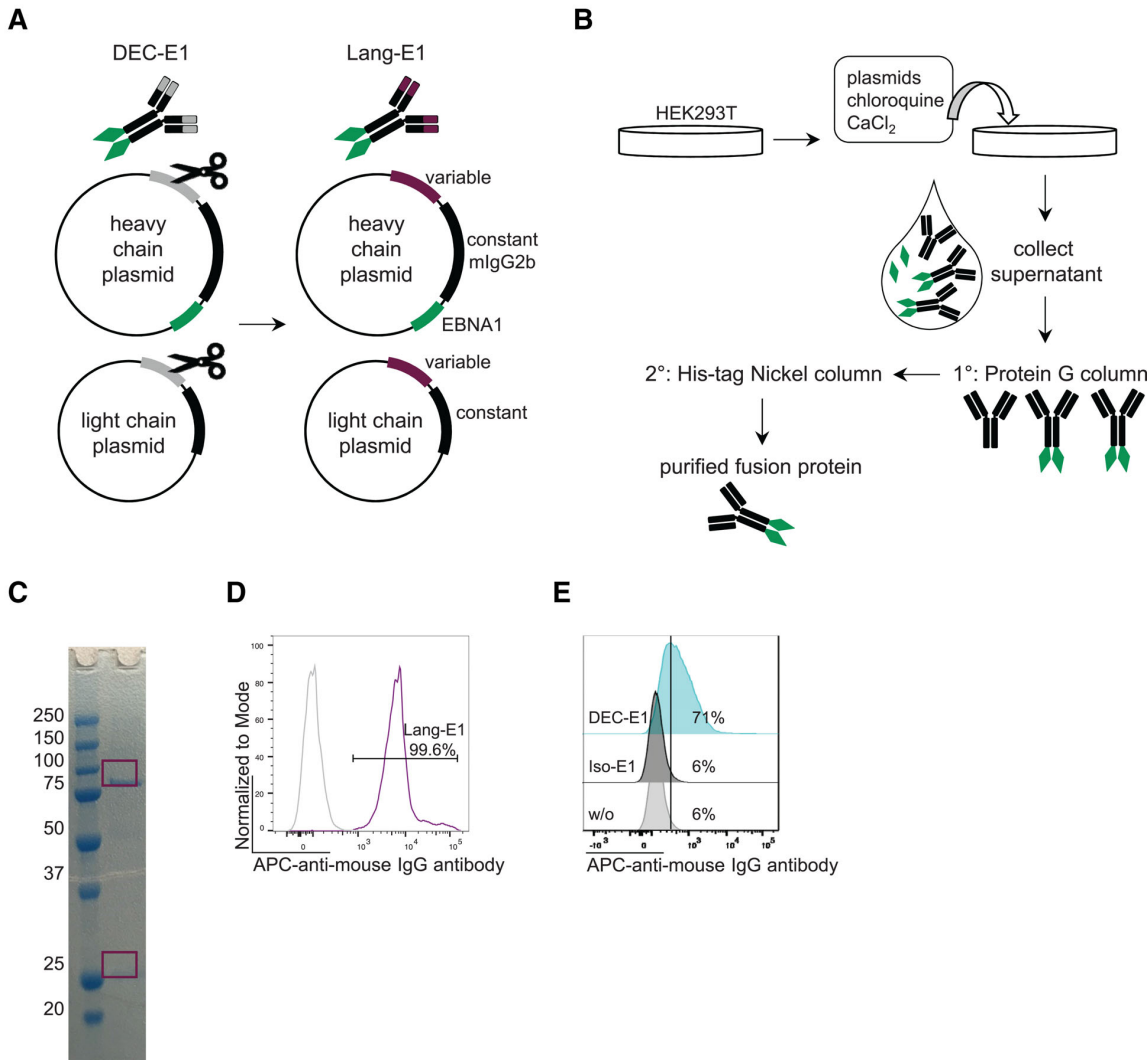
**Characterization of the anti‐Langerin‐EBNA1 fusion protein**. **(A)** Schematic illustration of the cloning procedure to obtain the anti‐Langerin‐EBNA1 fusion protein. Variable domains of the heavy and light chains of anti‐DEC‐205‐EBNA1 (DEC‐E1) were replaced with the variable domains from an anti‐Langerin antibody. **(B)** Schematic illustration of the different steps for fusion protein production. After calcium phosphate transfection of HEK293T cells supernatant was collected and applied to a two‐step FPLC purification with a protein G column followed by His‐tag Nickel column. **(C)** Reduced SDS‐PAGE gel of purified anti‐Langerin‐EBNA1 (Lang‐E1) fusion protein shows correct size: 80 kDa heavy chain+EBNA1, 25 kDa light chain. **(D)** The binding of anti‐Langerin‐EBNA1 (Lang‐E1) to human LC in epidermal cell suspension was detected with a fluorchrome‐conjugated secondary goat anti‐mouse IgG antibody by flow cytometry. LC were identified and gated as CD45^+^CD1a^+^HLA‐DR^+^ cells (gating strategy shown in Supporting information Figure S2). **(E)** The binding of anti‐DEC‐205‐EBNA1 (DEC‐E1) versus Isotype‐EBNA1 (iso‐E1) to immature monocyte‐derived DC was determined by detection with a secondary Ab as above. Representative results for at least three experiments.

Besides targeting DEC‐205 on skin DC, we wanted to address the targeting of the CLR Langerin. As antigen, the EBNA1 protein was used which allows for a functional read‐out of memory T cell responses from serologically EBV‐positive donors, which represent the majority of the human population [31]. The idea was to produce an anti‐Langerin‐EBNA1 fusion protein, which consists of the anti‐Langerin antibody genetically fused to the immunogenic C‐terminal domain of the EBNA1 protein (aa 400–641). For that purpose, the variable domains in the heavy chain and in the light chain plasmids of the anti‐DEC‐205‐EBNA1 fusion protein were replaced with the variable domains from an anti‐Langerin antibody (Fig. 1A). The Langerin antibody sequences were obtained by screening and sequencing an in‐house grown hybridoma cell line by Fusion Antibodies (Belfast, UK).

After successful cloning, fusion proteins were produced by calcium‐phosphate transfection of HEK293T cells and subsequent purification by a two‐step Protein G and His‐tag Nickel column by FPLC (Fig. 1B, detailed description in the Materials and Methods part). The resulting fusion protein consisted of two 80 kDa heavy chain‐EBNA1 molecules (i.e., 50 kDa heavy chain plus 30 kDa EBNA1) and two 25 kDa light chain molecules as confirmed in a reduced SDS‐PAGE gel (Fig. 1C). Thus, the overall size of the anti‐Langerin‐EBNA1 fusion protein was approximately 210 kDa. Furthermore, for comparison and control purposes, anti‐DEC‐205‐EBNA1 fusion protein as well as isotype‐EBNA1 fusion protein (isotype antibody with specificity against murine MHC class II), were produced. The binding abilities to the specific CLR were confirmed by flow cytometry by incubating the anti‐Langerin‐EBNA1 fusion protein with isolated human LC and the anti‐DEC‐205‐EBNA1 with monocyte‐derived DC (moDC) followed by a secondary fluorochrome‐coupled goat‐anti‐mouse IgG antibody (Fig. 1D and E).

In conclusion, we were able to exchange the variable domains of the anti‐DEC‐205‐EBNA1 fusion protein with the sequence from the anti‐Langerin antibody, and this resulted in the successful cloning and production of the anti‐Langerin‐EBNA1 fusion protein.

### LC targeted with anti‐Langerin‐EBNA1 in vitro induce antigen‐specific CD4^+^ T‐cell responses

Next, we investigated the function of this newly generated LC‐based vaccine in cocultures of LC with autologous T cells from the same skin donor. To obtain LC, epidermal cell suspensions were prepared from healthy donor skin by enzymatic digestion. These epidermal cell suspensions contain mainly keratinocytes but also 0.5–2% LC. They were cultured with GM‐CSF for 48 h in the presence or absence of poly I:C as an adjuvant, as TLR3 agonists are already used in clinical trials [5]. The phenotypical analysis for maturation markers CD83 and CD86 revealed no additional activation of CD1a^+^ HLA‐DR^+^ LC as they matured spontaneously [32], regardless of whether poly I:C was present or not (Supporting information Figure S2). Besides the maturation marker expression, LC also expressed the inhibitory molecules PD‐L1 and, at lower levels, PD‐L2 after 48 h of culture with no changes by poly I:C addition (Supporting information Figure S2).

**Figure 2:**
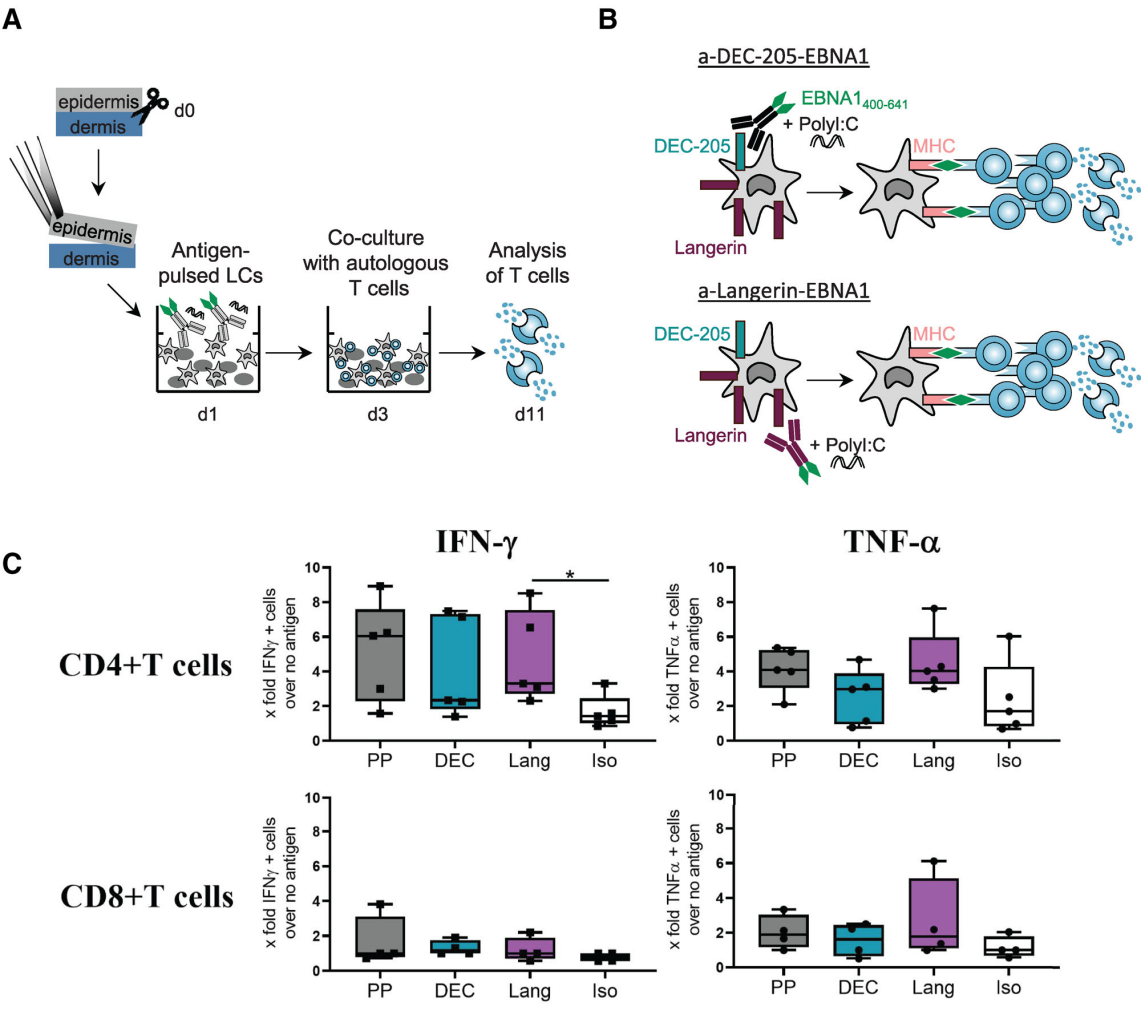
**LC targeted via Langerin in vitro induce EBNA1‐specific CD4^+^ T cell responses**. **(A and B)** Schematic illustration of targeting EBNA1 to DEC‐205 or Langerin on isolated LC with subsequent coculture with autologous CD4^+^ and CD8^+^ T cells. **(C)** LC were incubated for 48 h with 20 μg/mL poly I:C and either 1 μg/mL EBNA1‐targeting constructs/isotype control, or 1 μg/mL EBNA1 peptide pool. After coculture with autologous T cells for 8 days, T cells were restimulated for 6 h with EBNA1 peptide pool and analyzed for intracellular cytokines by flow cytometry. Fold increase of IFN‐γ^+^ cells and TNF‐α^+^ CD4^+^ and CD8^+^ T cells over “no antigen control” (LC cultured in medium only) is shown. Data from four to five donors combined from at least four independent experiments are shown. Mean ± SEM. *p < 0.05 as determined by unpaired two‐way ANOVA of log transformed data. PP…peptide pool, DEC…anti‐DEC‐EBNA1, Lang…anti‐Langerin‐EBNA1, Iso…isotype‐EBNA1.

To investigate the EBNA1‐specific T‐cell responses after targeting LC in the epidermal cell suspension, we incubated the cells for 48 h with 20 μg/mL poly I:C plus anti‐Langerin‐EBNA1. We compared this LC‐based vaccine with anti‐DEC‐205‐EBNA1 that was shown previously to induce T‐cell responses when targeted to moDC [33] as well as the isotype‐EBNA1, which would mimic a conventional immunization with untargeted antigen. As negative control, we cultured LC in medium without antigen, as positive control we loaded LC with EBNA1 peptide pool, both conditions in the presence of poly I:C. After 48 h, the mature, antigen‐loaded LC were cocultured with autologous PBMC from the same, serologically EBV‐positive, skin donor. These PBMC contain CD4^+^ and CD8^+^ T cells and allow simultaneous analysis of both T cell subtypes. After 8 days of coculture, cells were restimulated with the EBNA1 peptide pool for 6 h and intracellular production of cytokines was analyzed by flow cytometry (experimental set‐up illustrated in Fig. 2A and B). As exemplified in Supporting information Figure S3, we observed the induction of cytokines in T cells by targeting the model antigen EBNA1 to LC in vitro. Due to donor variations, for each donor, the percentages of the different conditions were normalized to the percentages of the “no antigen” control (LC incubated with medium plus poly I:C). Results are displayed as x‐fold ratio to “no antigen” control. We measured a two‐ to fourfold increase of IFN‐γ^+^ and TNF‐α^+^ CD4^+^ T cells over the isotype control when EBNA1 was targeted via Langerin or DEC‐205 to LC, and these results were statistically significant for IFNγ‐induction by Langerin targeting (Fig. 2C). EBNA1 peptide pool‐loaded LC served as a positive control and induced more cytokine‐producing CD4^+^ T cells (Fig. 2C). However, one has to keep in mind that the peptide pool cannot be stoichiometrically compared to the antigen in the fusion proteins. It was given in excess (1 μg/mL of each peptide), whereas in 1 μg/mL of fusion protein only 0.25 μg/mL EBNA1 protein is contained. We were not able to detect cytokine‐positive CD8^+^ T cells suggesting poor cross‐presentation of EBNA1 antigen by LC (Fig. 2C). This is most likely due to the low numbers of CD8^+^ T cell epitopes present in the EBNA1 protein antigen with specific HLA restrictions [31] and the unknown HLA subtype of the skin donors.

In conclusion, targeting EBNA1 to Langerin or DEC‐205 on LC activates functional cytokine‐producing CD4^+^ T cells, importantly, more than the identical amount of untargeted antigen (i.e., isotype fusion protein). Moreover, much less antigen is required to achieve similar T cell response when compared to peptide‐loaded LC.

### In contrast to DEC‐205, the intradermal application of anti‐Langerin‐EBNA1 does not activate T cells

The human skin explant model is a suitable tool to study antigen delivery to skin DC in their natural environment, which is of immense interest for translating knowledge into the clinics [27, 34, 35]. This model allows to investigate human skin DC function in a vaccination setting as close as possible to the in vivo situation. First of all, the phenotype of the spontaneously emigrated DC was analyzed after intradermal injection of poly I:C into skin explants. For this purpose, we prepared skin biopsies of 8 mm from healthy donor skin (Supporting information Figure S4A). After injection of 15 μg of the adjuvant poly I:C, skin explants were cultured on medium for 4 days and emigrated DC were analyzed by flow cytometry. Viable migratory skin immune cells, defined as CD45^+^HLA‐DR^+^ cells, are a mix of five different populations that can be distinguished as CD14^+^ macrophage like‐cells, CD1a^–^ cells, CD1a^+^ dermal DC (cDC2), CD141^+^ dermal DCs (cDC1), and CD1a^high^ LC. Due to their paucity, the cDC1 subset was not specifically studied here. Slightly more CD1a^+^ DC and less CD14^+^ cells emigrated after poly I:C injection compared to PBS injection; however, these results were not statistically different (Supporting information Figure S4B). Both CD1a^+^ cDC2 and LC displayed a mature phenotype with high expression of CD83 and expression of the inhibitory molecules PD‐L1 and PD‐L2, regardless of whether poly I:C was injected, indicative of spontaneous maturation during emigration (Supporting information Figure S4C).

**Figure 3:**
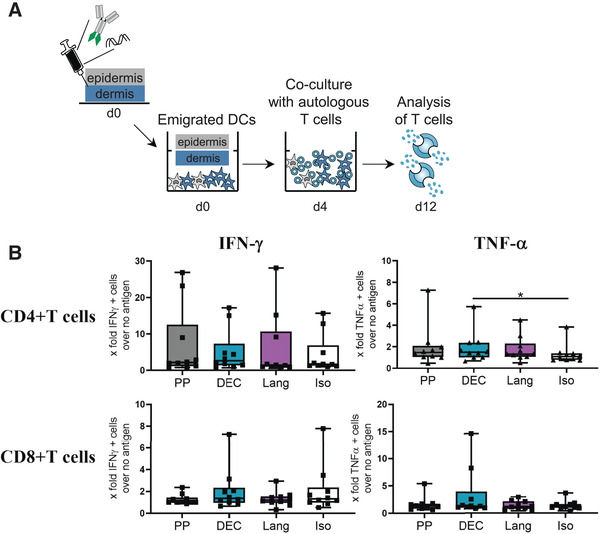
**Migratory LC targeted via Langerin in situ cannot stimulate T cells**. **(A)** Schematic illustration of intradermal injection of fusion proteins to target skin DC in situ, followed by coculture with autologous T cells. **(B)** Human skin explants were prepared as 8 mm punch biopsies and injected intradermally with 15 μg poly I:C and either 0.6 μg/mL EBNA1‐targeting constructs/isotype control, or 0.6 μg/mL EBNA1 peptide pool, or PBS (no antigen). The skin explants were cultured for 4 days and emigrated DC were cocultured with autologous CD4^+^ and CD8^+^ T cells at a DC:PBMC ratio of 1:40. After 8 days of coculture, cells were restimulated for 6 h with 1μg/mL EBNA1 peptide pool and analyzed for intracellular cytokine staining of CD4^+^ and CD8^+^ T cells by flow cytometry. Data from 10 donors combined from 10 independent experiments are shown. Mean ± SEM. *p < 0.05 as determined by unpaired two‐way ANOVA of log transformed data. PP…peptide pool, DEC…anti‐DEC‐EBNA1, Lang…anti‐Langerin‐EBNA1, Iso…isotype‐EBNA1.

To assess the potential of our novel LC‐based vaccine, we also investigated the T cell response after injection of the different targeting constructs together with poly I:C as the adjuvant into 8 mm skin biopsies. Spontaneously emigrated and antigen‐loaded skin DCs were collected after 4 days of skin explant culture. They were then cocultured with autologous PBMC in a ratio of 1:40 from the same skin donor. After 8 days of coculture, cells were restimulated with the EBNA1 peptide pool and analyzed for intracellular cytokine production by flow cytometry. The procedure is illustrated in Fig. 3A. As exemplified in Supporting information Figure S5, we observed induction of cytokines in T cells by targeting the model antigen EBNA1 to skin DC in situ. Again the percentages were normalized to the “no antigen” control (injection of PBS plus poly I:C) to circumvent donor variations. Results are displayed as x‐fold ratio to “no antigen” control. After analysis of multiple donors, we could detect significantly higher percentages of TNF‐α^+^ CD4^+^ T cells over the isotype control only when anti‐DEC‐EBNA1 was injected intradermally. All other comparisons revealed no differences between treatment groups (Fig. 3B).

**Figure 4:**
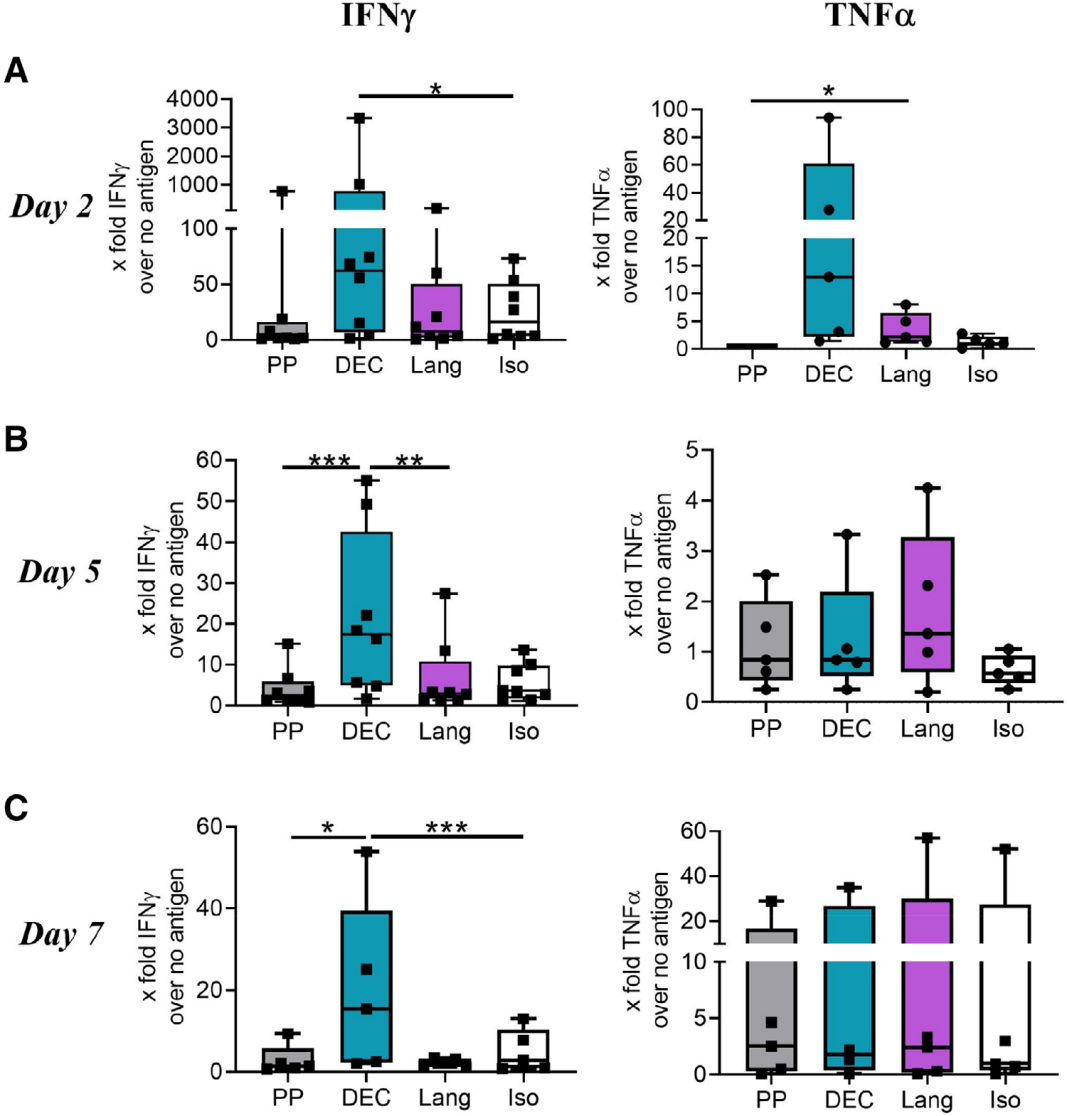
**Skin DC targeted via DEC‐205 in situ activate T cell cytokine release. (A‐C)** Supernatants from cocultures of migratory skin DC and autologous T cells were collected on days 2, 5, and 7. The levels of released IFN‐γ and TNF‐α were measured by Elisa on **(A)** day 2, **(B)** day 5, and **(C)** day 7 of coculture, Data from eight donors (IFN‐γ) and five donors (TNF‐α) combined from five to eight independent experiments are shown. Mean ± SEM. *p < 0.05, **p < 0.01, ***p < 0.001 as determined by two‐way ANOVA of log‐transformed data. PP…peptide pool, DEC…anti‐DEC‐EBNA1, Lang…anti‐Langerin‐EBNA1, Iso…isotype‐EBNA1.

EBNA1‐specific T cell responses are memory responses. Therefore, by day 8 of coculture, we might have missed the optimal time point for detection of these memory T cell cytokines, and so we also collected supernatants of cocultures and performed ELISA for TNF‐α and IFN‐γ at days 2, 5, and 7. Interestingly, at every time point, we detected statistically significant more IFN‐γ in culture supernatants when migratory DC were derived from skin explants that had been injected with anti‐DEC‐205‐EBNA1 but not with anti‐Langerin‐EBNA1 (Fig. 4A–C). In contrast, TNF‐α was only enhanced at day 2 of coculture after DEC‐205 targeting of skin DC, but again Langerin‐mediated targeting failed to induce cytokine production by T cells in coculture (Fig. 4A–C).

In conclusion, DEC‐205‐mediated targeting of the model antigen EBNA1 in skin explant cultures enabled migratory skin DC to activate autologous T cells although to a low level. In contrast, anti‐Langerin‐EBNA1 could not induce similar responses most likely due to the comparably lower numbers of emigrated LC from whole skin explants.

## Discussion

The emergence of immune checkpoint blockade antibodies was the major breakthrough in the field of cancer immunotherapy, with remarkable responses in a proportion of patients. However, severe side‐effects can occur, and immune resistance mechanisms are common. Therefore, checkpoint antibodies are tested not just as monotherapies but also as combination therapy [36]. A promising approach would be to use checkpoint antibodies together with DC‐based therapies as DC potently boost T cell responses or even induce de novo immune responses with minimal side‐effects [37]. In our study, we were interested in advancing our knowledge on DC‐targeting approaches, so we investigated the functional consequences of targeting antigens to human skin DC via the CLR DEC‐205 and Langerin. Addressing solely epidermal LC in vitro, we demonstrate that targeting antigen to Langerin or DEC‐205 with antibody‐antigen fusion proteins induced antigen‐specific CD4^+^ T cell responses that were somewhat stronger than by untargeted antigen. In situ in the human skin, we found that the delivery of DEC‐205‐targeted antigen, thereby, addressing LC and dermal DC, was more beneficial in the stimulation of antigen‐specific T cell responses than Langerin targeting of only LC. This suggests that targeting antigens to defined receptors on DC may enhance T cell immunity also in human skin, albeit not as pronounced as in mouse models. Furthermore, this emphasizes the importance of studying vaccination strategies not only with isolated cells but also with skin DC in their natural environment by using an ex vivo skin explant model that reflects the human situation as close as possible.

The special properties of DC to internalize, process, and present antigen and to prime naive T cells is exploited in vaccination approaches [4]. The two major strategies for DC‐based immunotherapy are ex vivo antigen loading of in vitro generated or isolated DC and in vivo targeting by surface receptors [3, 10]. For most clinical trials moDC were used, however, they may not be the best DC source and naturally occurring DC are currently tested [38]. Due to limitations regarding DC isolation and differentiation in vivo targeting approaches are developed to address DC in their natural environment [39]. As endocytic CLRs are expressed on defined DC subsets, their targeting results in addressing a vaccine to a single or multiple subsets [39, 40]. The human skin is an attractive vaccination site because it harbors a multitude of different DC subtypes with specialized functions [40, 41]. Indeed, mouse models showed that targeting skin DC enhances immune responses [14, 42, 43], and human skin DC can be targeted according to their CLR expression profile in an ex vivo skin explant model [27]. Regardless of the delivery mode, antibodies injected into the dermis diffused into the overlaying epidermis and were found in LC [27, 42]. In this study, we also demonstrate that for clinical purposes, the required human anti‐human antibodies against DEC‐205 bind to migratory LC and CD1a^+^ dermal DC, whereas an anti‐MR antibody preferentially targets CD14^+^ cells, which are more of a macrophage‐like cell type [30].

The right choice of adjuvant will be an important point in optimizing skin immunization with DC‐based immunotherapy. Early studies in mice showed that targeting DC without adjuvant‐induced tolerance instead of immunity [8], thus, for DC‐based cancer immunotherapy an effective adjuvant needs to be coadministered to achieve proper DC activation to strengthen the T‐cell response [3]. The TLR/RLR agonist poly I:C and its derivatives are frequently used in clinical trials and, via TLR3 or retinoic‐acid‐inducible protein 1/melanoma‐differentiation‐associated gene 5 (RIG‐I/MDA‐5) signaling induces activation of DCs [3]. The application of poly I:C had minor effects on maturation and migration of skin DC with a shift toward more CD1a^+^ DC and less CD14^+^ cells emigrating from skin explants. This is in line with observations that intradermal injection of TLR agonists or cytokines changes the distribution of migratory skin DC by increasing the number of dermal CD1a^+^ DC [27, 44]. In contrast to others [44], in our study, emigrated skin DC had the same expression levels of CD83 with or without poly I:C, indicating an already mature phenotype as a consequence of the migration process in skin explants. The surgery, skin explant preparation, and intradermal injection by themselves cause disturbance of the microenvironment leading to an inflammatory situation. The inhibitory ligands PD‐L1 and PD‐L2 were not further regulated by poly I:C injection as they are induced upon the migration process [45].

The major goal of this study was to fill the gap in knowledge on the relative capacities of the different human skin DC subsets to elicit an antigen‐specific T cell response after targeting a model antigen to CLR Langerin and DEC‐205. Therefore, we cloned and produced an LC‐based vaccine targeting Langerin by using an anti‐DEC‐205‐EBNA1 [33] as a backbone, and we exchanged the variable regions of the heavy and light chains with the Langerin‐binding sites. After successful production, we assessed the functional properties of our LC‐based vaccine by in vitro and in situ systems in comparison to the anti‐DEC‐205‐EBNA1 fusion protein [33]. By using skin and blood from serologically EBV‐positive healthy individuals, we were able to measure autologous memory T cell responses. Despite the fact that few EBNA1‐specific T cells are present in peripheral blood [31, 46], we were able to measure significantly higher IFN‐γ‐production in CD4^+^ T cells when they had been cocultured before with isolated LC loaded with anti‐Langerin‐EBNA1. We achieved similar response rates with anti‐DEC‐205‐EBNA1 and the positive control peptide pool. Remarkably, CD4^+^ T‐cell responses to fusion antibody were higher as compared to the identical amount of untargeted antigen (i.e., EBNA1 aa400‐641 fused to isotype control antibody). This is evidence that the enhancement of immune responses by targeting antigen to DC, as seen in mouse models [9, 11–13] may eventually be translated to humans. Variability between the donors was high indicating the different EBV‐memory T cell status. In addition, CD4^+^ T‐cell responses to the EBNA1 protein were easier to measure because more epitopes covering several HLA subtypes are present in the amino acid 400–641 stretch of the EBNA1 protein, fused to the antibodies, whereas just seven CD8^+^ epitopes with specific HLA restriction are expressed there [31]. This limits the possibility to investigate cross‐presentation by skin DC with the EBNA1‐model antigen.

Previous data demonstrated that anti‐DEC‐205‐EBNA1 induced EBNA1‐specific T cell responses [33, 47]. Furthermore, in line with our observations, EBNA1 stimulation in healthy human subjects resulted in activation of T‐cells producing cytokines [48, 49]. Due to functional differences between blood and skin DC [50], it is crucial to investigate human skin DC targeting approaches in their natural environment, for example, with the ex vivo skin explant model. When we applied anti‐DEC‐205‐EBNA1 fusion proteins in situ by intradermal injection into skin explants, we were able to detect cytokine production by T cells as measured by ELISA in culture supernatants. This was not the case with our LC‐based Langerin‐targeting vaccine, which can be explained by limitations of the skin explant model. Very few LC emigrate from whole‐skin explants and the bulk of migratory DC consists of CD1a^+^ dermal DC representing cDC2. These DEC‐205^+^ dermal DC outnumber LC in the presentation of antigen to T cells and to circumvent this problem sorting of migratory DC subsets would be required, a challenging task due to the low numbers of migratory DC per skin explant [34]. Interestingly, we found few cytokine‐positive T cells by flow cytometry indicative of poor antigen presentation by migratory skin DC. However, suboptimal timing of T cell restimulation with the peptide pool on day 8 of coculture could be another reason, and the detection of IFN‐γ in supernatants as early as day 2 of coculture supports this explanation. The choice of the receptor for targeting will also play a major role in determining the T‐cell response outcome. For example, Rühl et al. observed that DEC‐205 targeting gave the best results in terms of IFN‐γ secretion by CD4^+^ and CD8^+^ T‐cell clones compared to CD40 (mostly good CD4^+^ T‐cell response) or BDCA3‐targeting approaches (mostly good CD8^+^ T‐cell response); however, they did not investigate sole LC targeting [47]. Moreover, observations that engaging multiple DC subsets induced more potent T cell immunity [51, 52] speaks for DEC‐205 targeting of multiple skin DC subsets. This could indeed result in multifactorial T cell responses as LC cross‐present antigen for CTL response and CD1a^+^ dermal cDC2 prime CD8^+^ T cells and Th1 polarization [53, 54]. The isotype‐EBNA1 fusion protein used as a nontargeted antigen source elicited some T cell responses most likely by unspecific internalization mechanisms such as micropinocytosis, phagocytosis, or Fc‐receptor uptake. In fact, this isotype control may reflect the fate of any intradermally injected conventional, “untargeted” vaccine. Our results with the EBNA1 peptide pool are also of interest as peptides were given in excess. In vitro loaded isolated LC induced T cell responses, which is in line with Polak et al., who also found activation of T cells with EBV peptide‐pulsed LC in vitro [55]. In contrast, we observed that emigrated DC loaded in situ with the EBNA1 peptide pool did not induce a dominant T cell response; those loaded with the targeting anti‐DEC‐205‐EBNA1 construct did so, however. This confirms the advantage of targeted delivery of antigens over mere administration of peptides as also observed by others [9]. It is conceivable that other free protein/peptides might also compete for binding/uptake, might get degraded quickly, or bind to nonprofessional antigen presenting cells [39], all of this reducing the amount of antigen that becomes available for the skin DCs in situ.

In conclusion, DC are key coordinators in the immune system with a critical role for the induction of antitumor immunity. Insights on therapies based on DC, such as the targeting of CLR on human skin DC to elicit effective T cell immunity, are relevant for the integrated approach of future cancer therapies. Therefore, to empower the efficacy of DC‐based immunotherapies, it will be crucial to combine them with checkpoint blockade antibodies in the future. This will allow to unleash the full potential of DC‐based cancer vaccines by overcoming immunosuppression. We conclude that targeting skin DC holds potential for next‐generation vaccination approaches to enforce efficient anticancer immunity. As immune checkpoint inhibitor therapy is still limited to a subset of patients and primary and secondary resistance occurs, this could be beneficial for future combination therapy.

## Material and methods

### Human targeting antibodies

Human anti‐human DEC‐205/CD205 (clone 3G9, Celldex Therapeutics, NJ, USA), human anti‐human MR/CD206 (clone B11, Celldex) or respective isotype control (human IgG1, Southern Biotech, Alabama, USA) were used. These antibodies were obtained by immunizing mice transgenic for the human immunoglobulin, as described previously [21] and they were already applied—fused to the NY‐ESO‐1 tumor antigen [20] and named CDX‐1401‐ in the first clinical trials with melanoma patients [5, 23]. Human anti‐human targeting antibodies against DEC‐205 and MR were detected with a PE‐conjugated goat F(ab’)_2_ anti‐human IgG‐(γ) secondary antibody (Invitrogen).

### Cloning of the anti‐Langerin‐EBNA1 fusion protein

We used an already published anti‐DEC‐205‐EBNA1 vaccine [33] as a backbone for the cloning of the anti‐Langerin‐EBNA1 fusion protein. The heavy‐chain plasmid coding for anti‐DEC‐205‐EBNA1 was cut with the restriction enzymes EcoRI and BamHI (both New England Biolabs, USA) which cut around the spanning region of the variable domain resulting in a 6300 bp backbone heavy chain part without the variable region. The DNA from the 6300 bp backbone was purified with the PureLink Quick Gel Extraction kit (Thermo Fisher Scientific, USA) from a 1% agarose gel (Lonza, Switzerland). The sequence of the variable domain of the heavy chain of the anti‐Langerin antibody (Clone:DCGM4, derived from an in‐house‐cultured hybridoma cell line, a kind gift from Dr. J.J. Pin from Dendritics, France [now part of Eurobio‐Scientific, France]) was obtained from a monoclonal sequencing report carried out by Fusion Antibodies (Belfast, UK). This sequence, together with the spanning region containing the restriction enzyme sites for EcoRI and BamHI, was then ordered in a plasmid from GenScript (USA). This plasmid was cut and the small 580 bp insert DNA containing the variable domain of the heavy chain from the anti‐Langerin antibody was extracted and purified with the PureLink Quick Gel Extraction kit. The variable insert of the anti‐Langerin antibody and the backbone vector of the anti‐DEC‐205‐EBNA1 were ligated by using the Fast‐Link DNA ligation kit (Lucigen, USA) according to manufacturer's instructions. For the light chain, the procedure was similar: the light chain plasmid of the anti‐DEC‐205 antibody was cut with the restriction enzymes EcoRI and HindIII (New England Biolabs) resulting in a 4600 bp backbone part without variable region. The sequence of the variable domain of the light chain of the anti‐Langerin antibody was obtained from the same monoclonal sequencing report and purified as described above. The 750 bp insert containing the variable domain of the light chain of the anti‐Langerin antibody and the 4600 bp backbone vector of the anti‐DEC‐205 light chain were ligated by using the Fast‐Link DNA ligation kit according to manufacturers' instruction. Both ligation reactions were transformed in heat‐shock competent *E.coli* bacteria (Takara Bio Europe, France) according to manufacturers’ instruction. Small‐scale preparation of plasmid DNA was carried out by using the Plasmid Miniprep kit (Promega, USA) according to manufacturers instruction and checked for correct insertion by sequencing with MicroSynth (Switzerland). Preparation of plasmid DNA for transfection was carried out by using the Endofree Plasmid Maxi kit (Qiagen, Germany) according to manufacturers’ instructions.

### Production of the anti‐Langerin‐EBNA1 fusion protein

HEK293T cells (ATCC, No.CRL‐112689) were cultured for at least 1 week in medium consisting of DMEM high glucose (Sigma‐Aldrich, USA), 10% heat‐inactivated fetal‐calf serum (FCS, PAN‐Biotech, Germany), 4 mM l‐glutamine (Lonza), 200 U/mL penicillin, and 200 μg/mL streptomycin (Thermo Fisher Scientific), and 1 mM sodium pyruvate (Lonza). Addition of 1% nonessential amino acid (Thermo Fisher Scientific) and 1% Nutridoma (Roche, Switzerland) are optional. HEK293T cells were seeded in cell culture plates (Greiner Bio‐One, Austria) and treated with 20 mM Chloroquin (Sigma‐Aldrich) the next day prior to calcium‐phosphate transfection with plasmids encoding light and heavy chains of anti‐Langerin‐EBNA1 fusion protein. The day after transfection, the supernatant was carefully removed, the adherent HEK293T cells were washed with PBS (Thermo Fisher Scientific), and incubated for 2 days in a fresh medium consisting of DMEM high glucose, 4 mM l‐glutamine, 100 U/mL penicillin/100 μg/mL streptomycin, 1 mM sodium pyruvate, 1% nonessential amino acid, and 1% Nutridoma. The supernatant was collected, fresh medium was added to the cells for additional 3 days of culture. Supernatants from the two time points were pooled, centrifuged, sterile filtered (Stericup 0.22 μm; Merck, Germany), checked for a pH of 7, and stored at 4°C until further use.

### Purification of the anti‐Langerin‐EBNA1 fusion protein by FPLC

FPLC purification was carried out with the NGC chromatography system and fraction collector (Bio‐Rad Laboratories). The first purification was performed using a HiTrap Protein A HP column (GE Healthcare, USA) with 0.1 M glycine (pH 2, Carl Roth) as elution buffer in 500 μL fractions into tubes each containing 50 μL of 1 M Tris‐HCl (pH 8.8) to neutralize the pH value to 7. Fractions containing proteins, as verified by the high UV absorption signal from the FPLC, were pooled, diluted with PBS, and run over the second column, a HisTrap HP His‐tag Nickel column (GE Healthcare), with 500 mM imidazole (Sigma‐Aldrich) in PBS as elution buffer in 500 μL fractions. The fractions with the eluted protein were pooled, dialyzed overnight in a tube with 12–14 kDa cut‐off (Repligen, USA) against 5 L of cooled PBS. The protein concentration was determined by the Pierce BCA Protein Assay kit (Thermo Fisher Scientific) according to manufacturer's instruction. Endotoxin levels were quantified by using the Pierce LAL Chromogenic Endotoxin Quantitation kit (Thermo Fisher Scientific) and levels were routinely below 0.1 EU/μg.

### SDS‐Page gel of purified fusion protein

Protein samples were diluted with NuPage LDS sample buffer (Thermo Fisher Scientific) and 100 mM DTT (Sigma‐Aldrich) and boiled for 5 min at 95°C. As marker, the Odyssey Protein Molecular Weight Marker 928–4000 (LI‐COR) was used. The samples were loaded onto a NuPage 4–12% Bis‐Tris gel (Thermo Fisher Scientific) and run for 50 min at 180 V with the NuPage MOPS running buffer (Thermo Fisher Scientific). The gel was then stained with Instant Blue (Sigma‐Aldrich).

### Isolation of PBMC

Peripheral blood from healthy, human skin donors was obtained after written, informed consent and with approval of the local ethics committee (AN5003/2016 and AN5003/2019). Diluted blood (1:1 with PBS) was layered onto Lymphoprep (1.077 g/mL; STEMCELL Technologies, Canada) and PBMC were separated by density‐gradient centrifugation at 800 *g* for 30 min at room temperature. Plasma was obtained by centrifugation of undiluted blood and was sent for EBV antibody detection (EBV‐VCA‐IgG, EBV‐IgM, EBV‐EA, EBV‐NA) to the Institute of Virology, Medical University of Innsbruck, Austria to confirm that donors were serologically EBV positive (IgG^+^EBNA^+^). PBMCS were frozen in 90% FCS with 10% DMSO (Sigma‐Aldrich) for later use as a source of responder T cells for skin DC targeting experiments.

### Generation of monocyte‐derived DC (moDC)

CD14^+^ monocytes were isolated from PBMC by positive‐magnetic separation using anti‐human CD14‐magnetic particles (BD‐Biosciences) according to manufacturers’ instructions yielding a purity of over 95%. DCs were differentiated from monocytes by culture in medium consisting of RPMI‐1640 (PAN‐Biotech) with 2 mM l‐glutamine, 1× NEAA, 1% HEPES (Lonza), 1% heat‐inactivated human AB serum (PAN‐Biotech), and 50 μg/mL gentamicin (Gibco, Thermo Fisher Scientific) in a 6‐well plate (Falcon) together with 800 U/mL GM‐CSF (Leukine^®^, specific activity 5.6 × 10^6^ U/mg, Sanofi, France) and 500 U/mL IL‐4 (specific activity 5×10^6^ U/mg, PeproTech, USA). On day 3, 1 mL of the spent medium was replaced with 1.5 mL fresh medium containing 1000 U/mL IL‐4 plus 1600 U/mL GM‐CSF for an additional culture of 2 days to fully differentiate cells into immature moDC.

### Human skin samples

Healthy human skin samples were collected at the Department of Plastic, Reconstructive and Aesthetic Surgery, Medical University of Innsbruck, Innsbruck, Austria after informed consent and approval by the local ethics committee (AN5003/323/4.10 403/5.10 (4470a)). Skin was incubated in RPMI‐1640 complemented with 50 μg/mL gentamicin for 30 min for disinfection. Subcutaneous fat was removed with a scalpel or dermatome and skin pieces were further processed.

### Epidermal cell suspension

Skin pieces were incubated in RPMI‐1640 containing 1.5 U/mL dispase II (Roche) and 0.1% trypsin (Sigma‐Aldrich) overnight at 4°C followed by 30 min incubation at 37°C. Epidermis was peeled off the dermis with tweezers, broken up into smaller pieces, and pressed through a 100 μm cell strainer (Falcon) with a syringe plunger (BD Biosciences), followed by filtering through a 40 μm cell strainer (Falcon) to obtain a single‐cell suspension.

### Fusion protein‐binding assay

The binding of the fusion proteins was tested by incubating the different fusion proteins with either epidermal cells containing LC or with moDC for 15 min on ice. After washing, cells were stained with an APC‐conjugated secondary goat‐anti‐mouse IgG (Poly4053, BioLegend) for 15 min on ice and analyzed by flow cytometry.

#### Targeting LC/skin DC in epidermal cell suspensions in vitro or by intradermal injection in situ

Epidermal cells containing LC were incubated for 48 h with either 1 μg/ml of the fusion protein or 1 μg/mL of the EBNA1 peptide pool in the presence of 20 μg/mL poly I:C (Sigma‐Aldrich). The EBNA1 peptide pool consisted of a total of 51 overlapping (by 11 amino acids) peptides, 12 to 22 amino acids in length, of the B95.8 EBV strain [31] (peptides &elephants GmbH, Germany). The medium consisted of RPMI‐1640, 10% FCS, 2 mM l‐glutamine, and 50 μg/mL gentamicin, supplemented with 200 U/mL GM‐CSF (Biolegend). Human skin explants were prepared in the laboratory with 8 mm biopsy punches (Kai Medical Europe, Germany) and injected with 15 μg of poly I:C together with either 0.6 μg of the fusion protein or 0.6 μg of the EBNA1 peptide pool in 30 μL PBS intradermally with a 30G syringe (B Braun, Germany). Per condition, 5–10 biopsies were injected and placed onto a 100 μm cell strainer in 6 mL of RPMI‐1640 medium containing 10% FCS, 2 mM l‐glutamine, and 50 μg/mL gentamicin in a six‐well plate.

### Skin DC co‐culture with autologous T cells

Epidermal cell suspensions were washed after the 48 h loading period, counted, and numbers of LC determined based on their percentages assessed by flow cytometry. Most of the keratinocytes are dead and 0.5‐2 % LC can be detected in cell suspensions. The LC were cocultured with thawed, autologous PBMC at a LC:PBMC ratio of 1:40 in medium consisting of RPMI‐1640 with 1% human AB serum instead of FCS for 8 days at 37°C.

Migratory skin DC cells were harvested after 4 days, washed, and counted. Migratory skin DCs are mature and, therefore, easily recognizable as such by their pronounced cytoplasmic protrusions (“veils”) and, therefore, directly enumerable in the hemocytometer. They were also cocultured with thawed, autologous PBMC at a DC:PBMC ratio of 1:40 in the same medium as described above. On days 2, 5, and 7 of coculture, 100 μL of medium was removed for cytokine analysis and fresh medium containing 5 U/mL IL‐2 (Peprotech) was added. After 8 days of coculture, 1 μg/mL of EBNA1 peptide pool was added to all conditions for 6 h at 37°C. In the last 5 h, 1× Brefeldin A (Thermo Fisher Scientific) was added to stop cytokine secretion and cells were analyzed by flow cytometry.

### Flow cytometry

Flow cytometry experiments were performed on a FACS Canto II and analyzed with FlowJo 10 software (both BD Biosciences). Dead cells were excluded using the fixable viability‐dye‐eFluor780 (Thermo Fisher Scientific) and nonspecific FcR‐mediated staining was blocked with the human FcR blocking reagent (Miltenyi Biotec). Surface staining of cells was performed for 15 min at 4°C with fluorophore‐labeled antibodies as listed in Supporting information Table S1. Fluorescence Minus One or isotype‐matched antibodies were used as controls. For intracellular staining, the Cytofix/Cytoperm kit was used according to manufacturers’ instructions (BD Biosciences).

### Cytokine measurements

Supernatants from cocultures on days 2, 5 and 7 were used for OptEIA ELISA (BD Biosciences) for IFN‐γ and TNF‐α according to manufacturers’ instructions.

### Statistics

Data sets were tested for normality/lognormality using D'Agostino‐Pearson (datasets with n > 5) or Shapiro‐Wilk (datasets n ≤ 5) tests from GraphPad Prism software version 8 (GraphPad, San Diego, CA). Unpaired *t*‐tests, one‐ and two‐way ANOVA with Tukey's multiple comparisons test (parametric) were used as indicated in the figure legend. A *p‐*value of ≤0.05 was considered statistically significant (*p ≤ 0.01; **p ≤ 0.001; ***p ≤ 0.0001). If not explicitly indicated, differences were not statistically significant. Graphs were generated with GraphPad Prism software version 8.

## Conflict of interest

The authors declare no commercial or financial conflict of interest.

## Ethics statement

Healthy human skin samples were collected at the Department of Plastic, Reconstructive and Aesthetic Surgery, Medical University of Innsbruck, Innsbruck, Austria after informed consent and approval by the local ethics committee (AN5003/323/4.10 403/5.10 (4470a)).

## Authors’ Contribution

P.S. and N.R. developed the research project, acquired funding, and wrote the manuscript with support from L.B. The experiments were designed and performed by L.B., H.S., C.Z.‐R., and S. Sch., who also analyzed the data sets and prepared figures. Technical assistance was provided by D.O. and C.H. (cloning), C.H.T. (flow cytometry), S.S., J.R., G.C., and A.S. (fusion protein production), A.P. and S.Sp. (skin preparations), B.DF., S.Si., and J.K. (skin sample provision and preparation), T.K. and C.M. (fusion protein provision). Scientific advice was given by C.M. and T.K.

### Peer review

The peer review history for this article is available at https://publons.com/publon/10.1002/eji.202149670

**Abbreviations**
CLR: C‐type lectin receptors
MR: Mannose Receptor
cDC: conventional dendritic cells
LC: Langerhans cells
moDC: monocyte‐derived DCs
Bp: base pairs
FCS: Fetal calf serum

## Supporting information

Supporting Information

## Data Availability

The data that support the findings of this study are available from the corresponding author upon reasonable request.
