# Supplementary material for: Targeted delivery of a vaccine protein to Langerhans cells in the human skin via the C‐type lectin receptor Langerin
Source: Eur J Immunol. 2022 Jan 9;52(11):1829–41. doi: 10.1002/eji.202149670 (PMC9788233; doi:10.1002/eji.202149670)
Supplement: Supplementary file 1 — Supporting Information [file EJI-52-1829-s001.pdf]

Supplementary Figures:

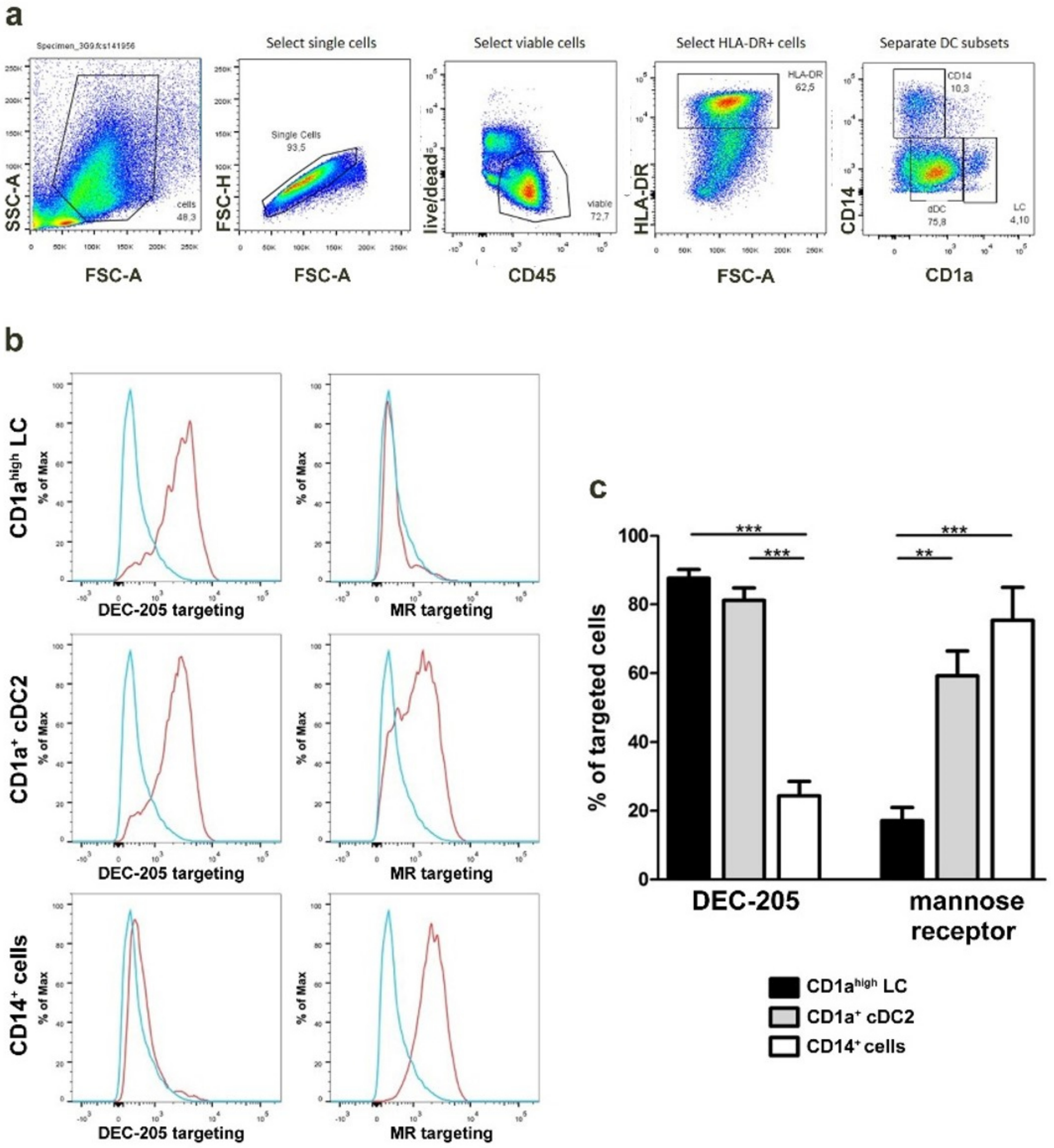

Supplementary Figure 1. Clinically applicable targeting antibody against DEC-205 or MR is transported by migratory LC and CD1a$^+$ dermal cDC2. Skin explants were intradermally injected with human anti-human DEC-205 (3G9) or human anti-human MR (B11) and cultured for 4 days. Emigrated cells were analysed for the expression of the bound targeting mAb by means of a fluorescent anti-human IgG antibody. (**a**) Gating strategy for migratory DC subsets is shown. (**b**) Histograms are representative for 7 experiments with skin from different donors. Blue line: injected isotype-matched control antibody; red line: injected anti-DEC-205 or anti-MR antibody. (**c**) Summary of up to 7 experiments for each condition is shown as percentage of targeted cells of each subset. Mean ± SEM. * $p < 0.05$, ** $p < 0.01$, *** $p < 0.001$ as determined by unpaired t-tests and 2-way ANOVA.

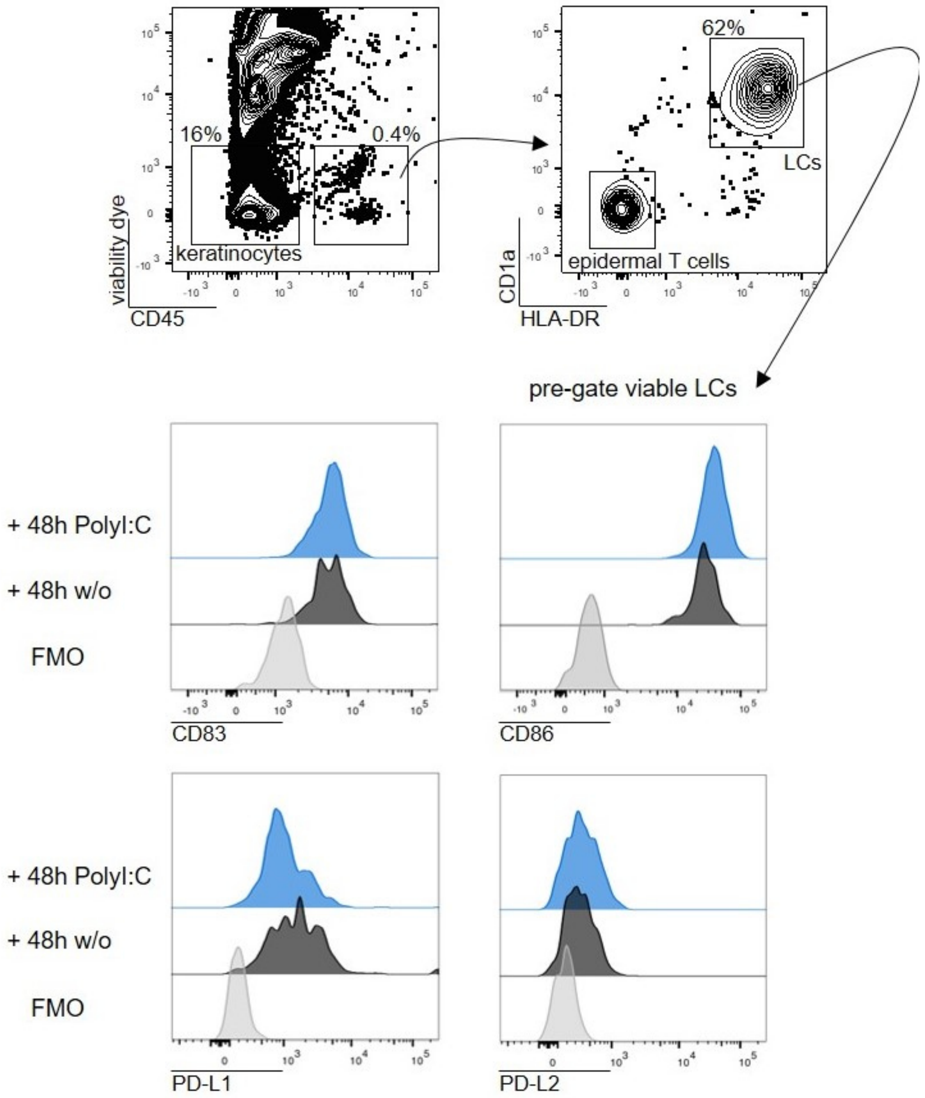

Supplementary Figure 2: LC activation unchanged by addition of polyI:C during *in vitro* culture. LC in epidermal cell suspension were analyzed after 48 h culture with GM-CSF plus/minus 20 µg/ml polyI:C for the expression of CD83, CD86, PD-L1, and PD-L2 by flow cytometry. LC were defined as viable $CD45^+CD1a^+HLA\text{-}DR^+$ cells. Representative plots for 4 donors from 4 experiments are shown.

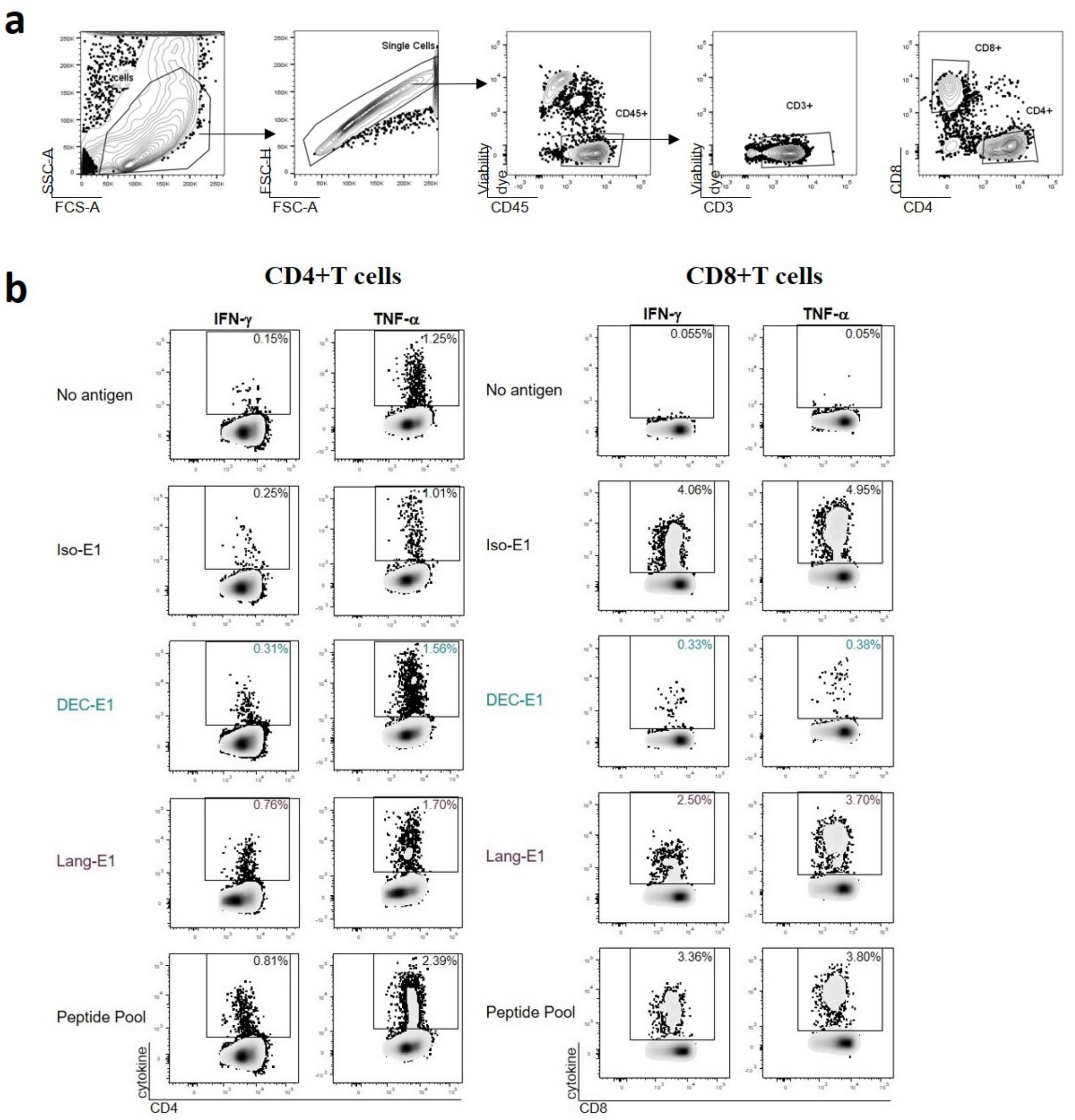

Supplementary Figure 3: Intracellular cytokine staining of T cells after targeting anti-DEC-205- and anti-Langerin-EBNA1 to human LC *in vitro*. LC were incubated for 48 h with 20 µg/ml polyI:C and either 1 µg/ml EBNA1-targeting constructs / isotype control, or 1 µg/ml EBNA1 peptide pool, or medium only (no antigen). LC were co-cultured with autologous T cells at a LC:PBMC ratio of 1:40. After 8 days of co-culture, cells were re-stimulated for 6h with 1 µg/ml EBNA1 peptide pool and analyzed for intracellular cytokine staining of $CD4^+$ and $CD8^+$ T cells by flow cytometry. Gating strategy is shown in (**a**), representative example out of 4-5 donors from at least 4 experiments is shown in (**b**).

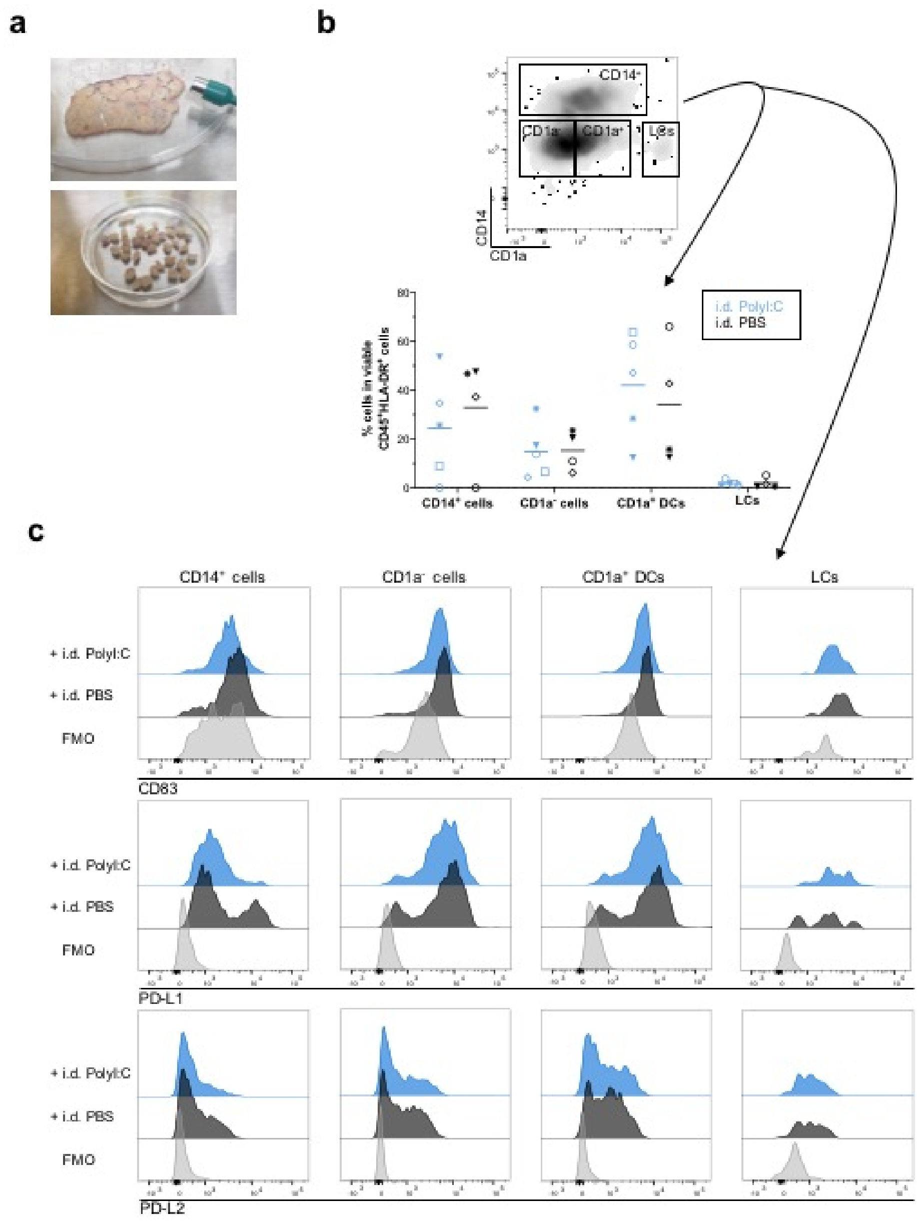

Supplementary Figure 4: Migratory skin DC activation unchanged by intradermal injection of polyI:C. (a) Human skin explants were prepared as 8 mm punch biopsies. **(b)** Skin biopsies were injected intradermally with 15 µg polyI:C and cultured for 4 days. The emigrated DC subsets were analyzed for their percentages within viable CD45^{+}HLA-DR^{+} cells. A summary graph from 4-5 donors from at least 4 experiments is shown. **(c)** Moreover, the expression of the maturation marker CD83 and the inhibitory molecules PD-L1 and PD-L2 were analyzed by flow cytometry. Representative histograms from 4-5 donors out of at least 4 experiments are shown.

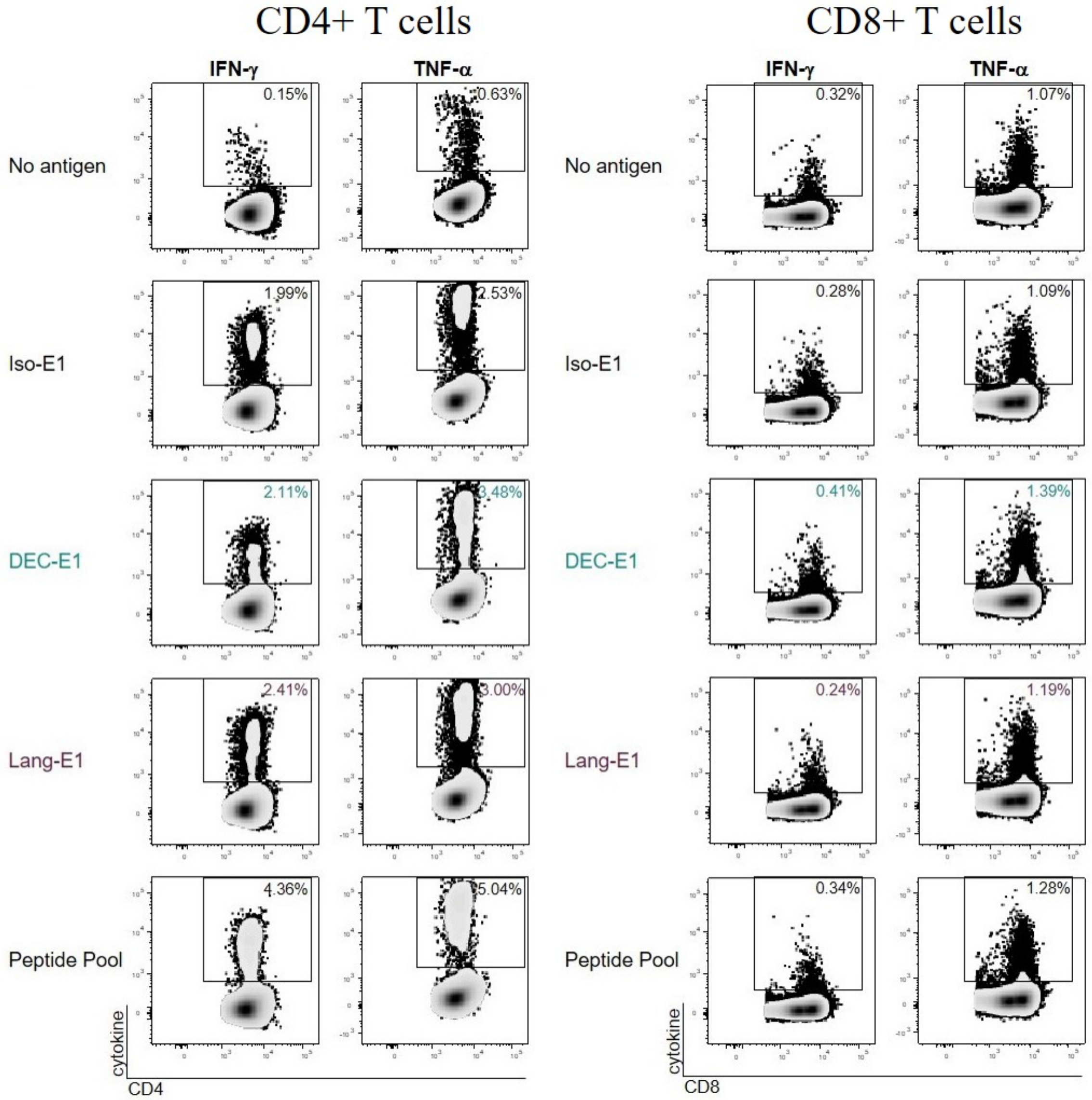

Supplementary Figure 5: Intracellular cytokine staining of T cells after targeting anti-DEC-205- and anti-Langerin-EBNA1 to human LC *in situ* in skin explants. Human skin explants were prepared as 8 mm punch biopsies and injected intradermally with 15 µg polyI:C and either 0.6 µg/ml EBNA1-targeting constructs / isotype control, or 0.6 µg/ml EBNA1 peptide pool, or PBS (no antigen). The skin explants were cultured for 4 days and emigrated DC were co-cultured with autologous T cells at a DC:PBMC ratio of 1:40. After 8 days of co-culture, cells were re-stimulated for 6h with 1 µg/ml EBNA1 peptide pool and analyzed for intracellular cytokine staining of $CD4^+$ and $CD8^+$ T cells by flow cytometry. Gating strategy is the same as shown in Supplementary Figure 3a, representative example out of 10 donors from 10 experiments is shown.

Supplementary table 1: antibodies used for flow cytometry

	Clone	Company
CD1a	HI149	BioLegend
CD4	SK3	BioLegend
CD8	RPA-T8	BioLegend
CD14	HCD14	BioLegend
CD45	HI30	BioLegend
CD83	HB15e	BioLegend
CD86	IT2.2	BD Biosciences
DEC-205	HD30	BioLegend
HLA-DR	L242	BioLegend
IFN-γ	4S.B3	BioLegend
Langerin	MB22-9F5	Miltenyi Biotec
PD-L1	MIH1	BD Biosciences
PD-L2	MIH18	BD Biosciences
TNF-α	MAb11	BioLegend
